# Low-Level Middle East Respiratory Syndrome Coronavirus among Camel Handlers, Kenya, 2019

**DOI:** 10.3201/eid2704.204458

**Published:** 2021-04

**Authors:** Peninah M. Munyua, Isaac Ngere, Elizabeth Hunsperger, Adano Kochi, Patrick Amoth, Lydia Mwasi, Suxiang Tong, Athman Mwatondo, Natalie Thornburg, Marc-Alain Widdowson, M. Kariuki Njenga

**Affiliations:** US Centers for Disease Control and Prevention, Nairobi, Kenya (P.M. Munyua, E. Hunsperger, M.A. Widdowson);; University of Nairobi, Nairobi (I. Ngere);; Washington State University, Nairobi (I. Ngere, M.K. Njenga)**;**; County Government of Marsabit, Marsabit, Kenya (A. Kochi);; Kenya Ministry of Health, Nairobi (P. Amoth, A. Mwatondo)**;**; Kenya Medical Research Institute, Nairobi (L. Mwasi);; US Centers for Disease Control and Prevention, Atlanta, Georgia, USA (S. Tong, N. Thornburg)

**Keywords:** MERS-CoV, asymptomatic, zoonoses, Middle East respiratory syndrome coronavirus, camels, viruses, viral zoonoses, Kenya, humans, infections

## Abstract

Although seroprevalence of Middle East respiratory coronavirus syndrome is high among camels in Africa, researchers have not detected zoonotic transmission in Kenya. We followed a cohort of 262 camel handlers in Kenya during April 2018–March 2020. We report PCR-confirmed Middle East respiratory coronavirus syndrome in 3 asymptomatic handlers.

Since the first human case of Middle East respiratory syndrome coronavirus (MERS-CoV) was identified in 2012, the World Health Organization has reported 2,494 infections and 858 deaths (case-fatality ratio 34.4%) in persons across 27 countries in the Middle East, Europe, Asia, and North America (*1*). Dromedary camels (*Camelus dromedarius*) are the known reservoirs of the virus (*2*,*3*). Most human cases result from direct or indirect transmission of virus from camels or human-to-human transmission in healthcare settings; researchers have also documented limited secondary transmission to household contacts (*4*). Occupational direct contact with camels is a risk factor for primary MERS-CoV infection (*5*). Camel workers and herders have a 0%–50% seroprevalence of MERS-CoV, generally higher than that of the general population in Saudi Arabia (*4*,*6*).

Although infection is widespread among dromedary camels, zoonotic transmission from camels to humans is sporadic, and disease prevalence among humans is not directly proportional to potential exposure to infected camels (*4*,*5*,*7*). Although >65% of the world’s dromedary camels live in Africa, on that continent MERS-CoV seroprevalence in humans is low (0.2%), with no documented cases of acute human infection (*8*,*9*). Furthermore, studies in the Africa region have identified MERS-CoV RNA in 11%–16% of camels and in 80%–95% of seropositive camels (*9*–*11*). To determine whether MERS-CoV infections occur in humans in a region with high seroprevalence among camels, we studied a cohort of 262 camel handlers in Kenya.

## The Study

**Figure 1:**
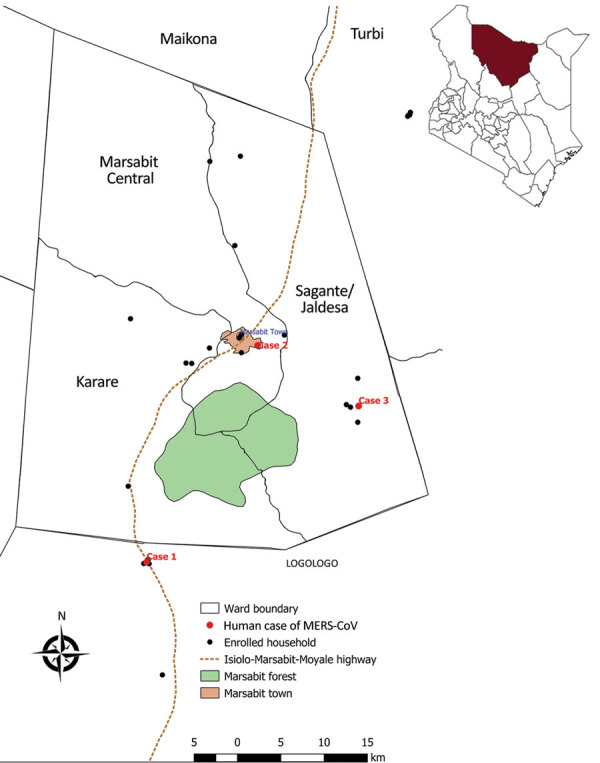
Locations of enrolled households in study on MERS-CoV, Marsabit County, Kenya, 2018–2020. Black circles indicate participating households; red circles indicate households with cases. Inset shows location of Marsabit County within Kenya. MERS-CoV, Middle East respiratory syndrome coronavirus.

**Figure 2:**
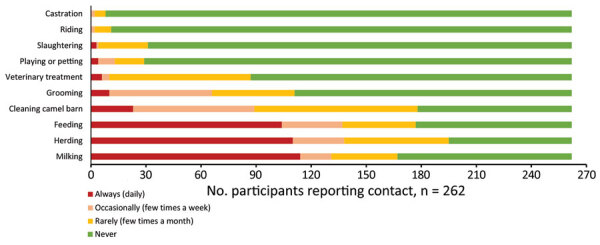
Types and frequency of contacts with camels among participants in study on Middle East respiratory syndrome coronavirus, Marsabit County, Kenya, 2018–2020.

During April 2018–March 2020, we enrolled participants on a rolling basis from 32 camel-owning households in Marsabit County, northern Kenya (Figure 1). We defined a camel handler as any person in the household who had contact with camels (Figure 2). This study was approved by the Scientific and Ethical Review Committee of Kenya Medical Research Institute (approval no. SSC3472), the Institutional Review Board of Washington State University (approval no. 16245), and the US Centers for Disease Control and Prevention (approval no. 7065). We obtained written informed consent from all participants.

We conducted monthly visits with the participants. At each visit, we collected nasopharyngeal and oropharyngeal swab samples from each participant. We also administered a questionnaire to each participating camel handler to identify signs and symptoms of possible respiratory illness during the previous 30 days. In addition, we recorded occurrences of respiratory illness among their household members. Participants belonged to 32 households with a median of 6 persons (interquartile range [IQR] 1–8 persons) and 32 camels (IQR 2–48 camels) at enrollment. The median age of these participants was 19 years (IQR 11–38 years). Most (67.2%) participants were male, of whom 39.3% were employed as camel workers and 38.2% were school going household members (Table 1). All participants handled camels. The most frequent interactions were herding (74.4%), cleaning barns (67.9%), feeding (67.6%), and milking (63.7%) (Figure 2).

**Table 1:** Characteristics of camel handlers enrolled in a study on Middle East respiratory syndrome coronavirus, Marsabit, Kenya, 2018–2020*

Characteristic	Value, n = 262
Sex	
M	176 (67.2)
F	86 (32.8)
Age, y	
<19	131 (50)
20–39	73 (27.9)
40–59	41 (15.6)
>60	17 (6.5)
Median participant age, y (IQR)	19 (11–38)
Work engagement in the previous 30 d	
Camel farm worker	103 (39.3)
Primary/secondary school student	100 (38.2)
Housewife	28 (10.7)
Farm owner	22 (8.4)
Pastoralist	6 (2.3)
Not currently engaged	2 (0.8)
Retiree	1 (0.4)
Median household size (IQR)	6 (1–8)
Median camel herd size (IQR)	32 (2–48)
History of chronic respiratory symptoms†	5 (1.9)
History of other chronic conditions‡	1 (0.4)
History of travel in the previous 5 y	0
Past tobacco use	72 (27.5)
Current tobacco use	72 (27.5)
Respiratory symptoms during the 2-year follow-up period	25 (9.5)
Fever	5 (1.9)
Running nose	39 (14.9)
Cough	25 (9.5)
Nasal stuffiness	23 (8.8)
Sore throat	16 (6.1)
Chest pain	2 (0.8)

*Values are no. (%) except as indicated. †Respiratory symptoms include cough, sore throat, running nose, nasal stuffiness, chest pain. ‡Other chronic medical conditions include diabetes, cancer, liver disease, kidney disease, and tuberculosis.

We stored the swab samples in virus transport media and tested them for MERS-CoV by reverse transcription PCR (RT-PCR) at the Kenya Medical Research Institute (Nairobi, Kenya) as described previously (*12*). We conducted real-time RT-PCR selective for the upstream region envelope and 2 distinct regions of nucleocapsid genes on total nucleic acid extracted from 200 μL of sample. We defined a positive sample by positivity of all 3 PCRs.

We tested 1,369 samples from 262 camel handlers during the 2-year follow-up period. Participants had a median of 43.6% of monthly follow-up visits (IQR 8%–75%). Three (1.1%) participants (cases 1–3) tested positive for MERS-CoV by RT-PCR. The cycle threshold (C_t_) values for case 1 were 38.9 for the upstream envelope, 37.7 for the nucleocapsid 2, and 39.3 for the nucleocapsid 3 genes; for case 2, the values were 39.7 for the upstream envelope, 36.9 for the nucleocapsid 2, and 39.8 for the nucleocapsid 3 genes; for case 3, the values were 35.6 for the upstream envelope, 36.0 for the nucleocapsid 2, and 36.8 for the nucleocapsid 3 genes. We detected all 3 cases during July–September 2019 (Table 2). 

**Table 2:** Characteristics of persons with Middle East respiratory syndrome coronavirus, Kenya, 2019

Case	Age, y/sex	Occupation	No. household members in study	No. of camels owned by household	Enrollment date	Diagnosis date	Type of camel contact at time of positive sample
1	20/F	Spouse to herder	11	37	2019 Jun 23	2019 Jul 31	Herding, milking, feeding, petting/playing, grooming, cleaning barn
2	49/M	Herder	6	2	2019 May 21	2019 Aug 13	Herding, milking, feeding, grooming, cleaning barn, administering medications
3	22/M	Herder	3	32	2018 May 2	2019 Sep 12	Herding, milking, feeding, grooming, cleaning barn, assisting in birth

Case 1 was in a woman 20 years of age who enrolled in June 2019 and had 9 monthly follow-up visits. She participated in the study with 11 other members of the household, all of whom tested negative for MERS-CoV throughout the follow-up period. Case 2 was in a man 49 years of age who enrolled in May 2019 and had 7 monthly follow-up visits. He participated in the study with 6 of his 10 household members; all the participants in his household tested negative for MERS-CoV. Case 3 was in a man 22 years of age who enrolled in May 2018 and had 12 monthly follow-up visits. He participated in the study with 3 of his 9 household members; the participants in his household tested negative for MERS-CoV. None of the 3 with positive results tested positive for MERS-CoV in the subsequent months.

All of the 3 with positive results were asymptomatic at diagnosis and had no concurrent conditions or histo-ry of travel outside of the county or country in the previ-ous month. None of them or their household members had respiratory illness before or after diagnosis.

## Conclusions

We report 3 PCR-confirmed cases of MERS-CoV in humans in Kenya; these cases met the World Health Organization case definition of MERS-CoV infection (*13*). All 3 persons were asymptomatic before and after diagnosis; this finding supports previous data suggesting that the virus causes no or mild disease in Africa compared with the Middle East and Asia, perhaps because of the younger age of most camel herders in Africa (*4*,*8*,*9*). Our findings are limited by the high C_t_ values (>35) of all cases, a level which some experts might not consider to be positive. However, because these patients had C_t_ values <40 for 3 distinct MERS-CoV genes, we feel confident that these are unlikely to be false positive results. Researchers have observed low upper respiratory tract RNA concentrations in asymptomatic patients and contacts of MERS-CoV patients (*14*). In contrast to studies conducted in the Middle East, we found no evidence of human-to-human transmission; a total of 20 household members of the 3 patients tested negative for MERS-CoV before and after their household member’s diagnosis. However, we might have missed some infections that occurred between follow-up visits. Furthermore, not all household members were enrolled in the study. In addition, serologic assessment of MERS-CoV T-cell responses might detect mild and asymptomatic MERS-CoV cases (*15*). Finally, the low (0.2%) seroprevalence among participants who had high exposure to camel herds with MERS-CoV circulation suggest a low level of zoonotic camel-to-human transmission. We previ-ously found no antibodies against MERS-CoV in camel herders despite high seroprevalence among camels in this community (*9*). 

In conclusion, we confirmed zoonotic transmis-sion of MERS-CoV from camels to handlers in Kenya. Focused surveillance is needed to detect these rare in-fections when they occur.

## References

[1] World Health Organization. Middle East respiratory syndrome coronavirus (MERS-CoV) summary updates, November 2019. 2019 [cited 2020 Jul 6]. https://www.who.int/emergencies/mers-cov/en

[2] Ali MA, Shehata MM, Gomaa MR, Kandeil A, El-Shesheny R, Kayed AS, et al. Systematic, active surveillance for Middle East respiratory syndrome coronavirus in camels in Egypt. Emerg Microbes Infect. 2017;6:e1. 10.1038/emi.2016.130 28050021 PMC5285495

[3] Reusken CB, Farag EA, Jonges M, Godeke GJ, El-Sayed AM, Pas SD, et al. Middle East respiratory syndrome coronavirus (MERS-CoV) RNA and neutralising antibodies in milk collected according to local customs from dromedary camels, Qatar, April 2014. Euro Surveill. 2014;19:20829. 10.2807/1560-7917.ES2014.19.23.20829 24957745

[4] Grant R, Malik MR, Elkholy A, Van Kerkhove MD. A review of asymptomatic and subclinical Middle East respiratory syndrome coronavirus infections. Epidemiol Rev. 2019;41:69–81. 10.1093/epirev/mxz009 31781765 PMC7108493

[5] Hui DS, Azhar EI, Kim YJ, Memish ZA, Oh MD, Zumla A. Middle East respiratory syndrome coronavirus: risk factors and determinants of primary, household, and nosocomial transmission. Lancet Infect Dis. 2018;18:e217–27. 10.1016/S1473-3099(18)30127-0 29680581 PMC7164784

[6] Alshukairi AN, Zheng J, Zhao J, Nehdi A, Baharoon SA, Layqah L, et al. High prevalence of MERS-CoV infection in camel workers in Saudi Arabia. MBio. 2018;9:e01985-18. 10.1128/mBio.01985-18 30377284 PMC6212820

[7] Hemida MG, Al-Naeem A, Perera RA, Chin AW, Poon LL, Peiris M. Lack of middle East respiratory syndrome coronavirus transmission from infected camels. Emerg Infect Dis. 2015;21:699–701. 10.3201/eid2104.141949 25811546 PMC4378477

[8] Liljander A, Meyer B, Jores J, Müller MA, Lattwein E, Njeru I, et al. MERS-CoV antibodies in humans, Africa, 2013–2014. Emerg Infect Dis. 2016;22:1086–9. 10.3201/eid2206.160064 27071076 PMC4880087

[9] Munyua P, Corman VM, Bitek A, Osoro E, Meyer B, Müller MA, et al. No serologic evidence of Middle East respiratory syndrome coronavirus infection among camel farmers exposed to highly seropositive camel herds: a household linked study, Kenya, 2013. Am J Trop Med Hyg. 2017;96:1318–24. 10.4269/ajtmh.16-0880 28719257 PMC5462565

[10] Chu DKW, Hui KPY, Perera RAPM, Miguel E, Niemeyer D, Zhao J, et al. MERS coronaviruses from camels in Africa exhibit region-dependent genetic diversity. Proc Natl Acad Sci U S A. 2018;115:3144–9. 10.1073/pnas.1718769115 29507189 PMC5866576

[11] Corman VM, Jores J, Meyer B, Younan M, Liljander A, Said MY, et al. Antibodies against MERS coronavirus in dromedary camels, Kenya, 1992-2013. Emerg Infect Dis. 2014;20:1319–22. 10.3201/eid2008.140596 25075637 PMC4111164

[12] Corman VM, Ölschläger S, Wendtner CM, Drexler JF, Hess M, Drosten C. Performance and clinical validation of the RealStar MERS-CoV Kit for detection of Middle East respiratory syndrome coronavirus RNA. J Clin Virol. 2014;60:168–71. 10.1016/j.jcv.2014.03.012 24726679 PMC7106532

[13] World Health Organization. Middle East respiratory syndrome case definition for reporting to WHO. 2017 [cited 2020 Oct 2]. https://www.who.int/csr/disease/coronavirus_infections/mers-interim-case-definition.pdf

[14] Drosten C, Meyer B, Müller MA, Corman VM, Al-Masri M, Hossain R, et al. Transmission of MERS-coronavirus in household contacts. N Engl J Med. 2014;371:828–35. 10.1056/NEJMoa1405858 25162889

[15] Mok CKP, Zhu A, Zhao J, Lau EHY, Wang J, Chen Z, et al. T-cell responses to MERS coronavirus infection in people with occupational exposure to dromedary camels in Nigeria: an observational cohort study. Lancet Infect Dis. 2020;S1473-3099(20)30599-5. 10.1016/S1473-3099(20)30599-5 33035474 PMC7538089

